# Role of glycosylphosphatidylinositol‐anchored high‐density lipoprotein binding protein 1 in hypertriglyceridemia and diabetes

**DOI:** 10.1111/jdi.14056

**Published:** 2023-07-13

**Authors:** Naoko Kurooka, Jun Eguchi, Jun Wada

**Affiliations:** ^1^ Department of Nephrology, Rheumatology, Endocrinology and Metabolism, Faculty of Medicine, Dentistry and Pharmaceutical Sciences Okayama University Okayama Japan

**Keywords:** Dibaetes, GPIHBP1, Lipoprotein lipase

## Abstract

In diabetes, the impairment of insulin secretion and insulin resistance contribute to hypertriglyceridemia, as the enzymatic activity of lipoprotein lipase (LPL) depends on insulin action. The transport of LPL to endothelial cells and its enzymatic activity are maintained by the formation of lipolytic complex depending on the multiple positive (glycosylphosphatidylinositol‐anchored high‐density lipoprotein binding protein 1 [GPIHBP1], apolipoprotein C‐II [APOC2], APOA5, heparan sulfate proteoglycan [HSPG], lipase maturation factor 1 [LFM1] and sel‐1 suppressor of lin‐12‐like [SEL1L]) and negative regulators (APOC1, APOC3, angiopoietin‐like proteins [ANGPTL]3, ANGPTL4 and ANGPTL8). Among the regulators, GPIHBP1 is a crucial molecule for the translocation of LPL from parenchymal cells to the luminal surface of capillary endothelial cells, and maintenance of lipolytic activity; that is, hydrolyzation of triglyceride into free fatty acids and monoglyceride, and conversion from chylomicron to chylomicron remnant in the exogenous pathway and from very low‐density lipoprotein to low‐density lipoprotein in the endogenous pathway. The null mutation of GPIHBP1 causes severe hypertriglyceridemia and pancreatitis, and GPIGBP1 autoantibody syndrome also causes severe hypertriglyceridemia and recurrent episodes of acute pancreatitis. In patients with type 2 diabetes, the elevated serum triglyceride levels negatively correlate with circulating LPL levels, and positively with circulating APOC1, APOC3, ANGPTL3, ANGPTL4 and ANGPTL8 levels. In contrast, circulating GPIHBP1 levels are not altered in type 2 diabetes patients with higher serum triglyceride levels, whereas they are elevated in type 2 diabetes patients with diabetic retinopathy and nephropathy. The circulating regulators of lipolytic complex might be new biomarkers for lipid and glucose metabolism, and diabetic vascular complications.

## Graphical abstract

Glycosylphosphatidylinositol anchored high density lipoprotein binding protein 1 (GPIHBP1) is a crucial molecule for the translocation of lipoprotein lipase from parenchymal cells to luminal surface of capillary endothelial cells, and maintenance of lipolytic activity. The null mutation of GPIHBP1 and the production of GPIHBP1 autoantibody induce severe hypertriglyceridemia and recurrent episodes of acute pancreatitis. In patients with type 2 diabetes, the circulating GPIHBP1 levels might not correlate well with triglyceride‐rich lipoproteins, and rather well‐reflect the microvascular complications, such diabetic retinopathy and diabetic kidney disease.

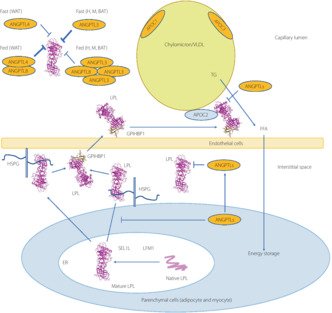

## INTRODUCTION

The elevation of triglyceride (TG)‐rich lipoproteins (TRL), such as chylomicrons, very low‐density lipoproteins (VLDL) and their remnants, plays a causal role in the development of atherosclerotic cardiovascular disease (ASCVD)^1^. In both type 1 and 2 diabetes patients, reduced or deficient insulin secretion and insulin resistance contribute to hypertriglyceridemia, as the enzymatic activity of lipoprotein lipase (LPL) depends on insulin action. In the management of hypertriglyceridemia in diabetes, statin remains the first‐line pharmacological therapy after lifestyle optimization, such as elimination of added sugars, abstinence from alcohol, limitation of total fat intake to 10–15% of the caloric intake, and aerobic physical activity for the primary and secondary prevention of ASCVD. Among the second‐line medications, omega‐3 fatty acids showed the reduction of ASCVD in the Japan EPA Lipid Intervention Study (JELIS)^2^ and the Reduction of Cardiovascular Events with Icosapent Ethyl–Intervention Trial (REDUCE‐IT)^3^. However, there were also conflicting results in the clinical trials that failed to show cardiovascular benefits in A Study of Cardiovascular Events in Diabetes (ASCEND) trial^4^ and the Statin Residual Risk Reduction with Epanova in High Cardiovascular Risk Patients with Hypertriglyceridemia (STRENGTH)^5^. Fibrates activate peroxisome proliferator‐activated receptor‐α and increase the activity of LPL, and they are also the second‐line medications potentially preventing ASCVD in patients with diabetes and hypertriglyceridemia. In the Fenofibrate Intervention and Event Lowering in Diabetes (FIELD) study^6^ and the Action to Control Cardiovascular Risk in Diabetes.

(ACCORD) Lipid study^7^, fenofibrate failed to show cardiovascular risk reduction and was no longer used as an ASCVD prevention medication. Recently, pemafibrate, a selective peroxisome proliferator‐activated receptor‐α modulator, also failed to lower the incidence of cardiovascular events in patients with type 2 diabetes, mild‐to‐moderate hypertriglyceridemia (TG level 200–499 mg/dL) and high‐density lipoprotein (HDL) cholesterol levels of ≤40 mg/dL^8^.

**Figure 1:**
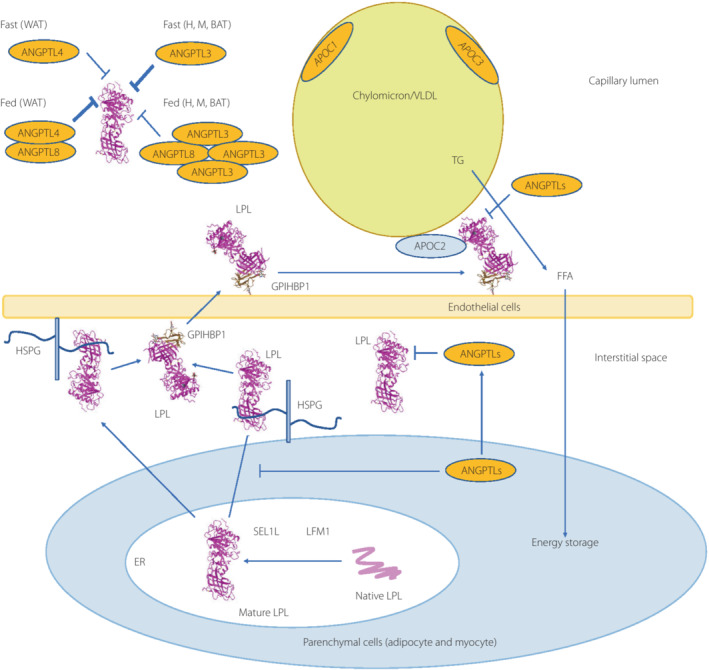
Lipoprotein lipase (LPL) complex bound to capillary endothelial cell surface. LPL bound to endothelial cells is a key enzyme for hydrolyzation of triglyceride (TG) into free fatty acids (FFA) and monoglyceride. Head‐to‐tail homodimer formation was thought to be critical for LPL secretion and the maintenance of the enzymatic activity, whereas recent data suggested that LPL is synthesized and secreted as a monomer, and chaperoned all the way through the biosynthesis pathway to maintain its mature structure. During the biosynthesis of LPL in parenchymal cells, such as adipocyte and myocyte, LPL is chaperoned by lipase maturation factor 1 (LMF1) and Sel‐1 suppressor of Lin‐12‐Like 1 (SEL1L), and secreted through the trans‐Golgi network (pink ribbon model). LPL next binds to heparan sulfate proteoglycan, and is stabilized in the extracellular matrix in the interstitial space, and glycocalyx on the endothelial cells and parenchymal cells. LPL next forms the complex with glycosylphosphatidylinositol‐anchored high‐density lipoprotein binding protein 1 (GPIHBP1; brown ribbon model), and they are shuttled to the capillary lumen of the endothelial cells. Apolipoprotein C‐II (APOC2) on the chylomicron and very low‐density lipoprotein is a critical factor for LPL hydrolyzation enzymatic activity, whereas APOC1 and APOC3 compete with LPL for binding to lipid emulsion particles. The binding of angiopoietin‐like protein (ANGPTL)4 to LPL adjacent to the catalytic cavity triggers the unfolding of LPL's hydrolase domain, resulting in irreversible collapse of the catalytic cavity and loss of LPL activity. ANGPTL3– ANGPTL8 regulates the lipid intake in the heart (H), skeletal muscle (M) and brown adipose tissue (BAT) by the inhibition of LPL in the fed state, whereas ANGPTL8 in white adipose tissues (WAT) attenuates LPL inhibition by ANGPTL4 to promote triglyceride (TG)‐rich lipoprotein processing. ER, endoplasmic reticulum.

LPL bound to endothelial cells is a key enzyme for hydrolyzation of TG into free fatty acids and monoglyceride, and critically involved in conversion from chylomicron (CM) to CM remnant in the exogenous pathway and from VLDL to LDL in the endogenous pathway^9^. The lipolytic activity of LPL depends on the multiple positive and negative regulators forming a complex on the endothelial cells. In the parenchymal cells, lipase maturation factor 1 (LMF1) dimerizes and activates LPL, and a specific chaperone, namely, Sel‐1 suppressor of Lin‐12‐Like 1 (SEL1L), stabilizes the LPL–LMF1 complex. Subsequently, LPL released from parenchymal cells is captured by heparan sulfate proteoglycan within the subendothelial spaces, and translocated to the endothelial cell luminal surface of capillaries by binding to glycosylphosphatidylinositol‐anchored high‐density lipoprotein‐binding protein (GPIHBP1). Apolipoprotein C‐II (APOC2) is a critical factor for LPL activity and APOA5 stabilized LPL‐APOC2 complex, while APOC1 and APOC3 compete with LPL for binding to lipid emulsion particles^10^. Furthermore, angiopoietin‐like proteins (ANGPTL) 3, 4, and 8 are also described to inhibit LPL^11^ (Figure 1). In this review, we focus on the recently identified GPIHBP1, and describe the biological function, relevance in hypertriglyceridemia and new insights in the diabetic vascular complications.

## ROLE OF GPIHBP1 IN TG‐RICH LIPOPROTEIN PROCESSING

GPIHBP1 was originally identified during the screening of a mouse liver complementary deoxyribonucleic acid library by the binding activity to HDL^12^. However, in later studies, GPIHBP1 was shown to bind LPL and APOA5, but not bind APOA1 and HDL^13^. The important discovery was that the chow‐fed *Gpihbp1* knockout mice (*Gpihbp1* ^−/−^) showed severe hypertriglyceridemia with plasma TG levels ranging from 2,500 to 3,500 mg/dL^14^, ^15^, ^16^ and markedly reduced LPL in the post‐heparin plasma, suggesting that GPIHBP1 plays a critical role in LPL‐mediated TG‐rich lipoprotein processing. The tissue distribution of GPIHBP1 is like LPL, and highly expressed and confined to the endothelial cells of capillaries in the heart, skeletal muscle and adipose tissues with active TG‐rich lipoprotein processing, except the lung with high levels of GPIHBP1 expression, but extremely low expression of LPL^14^. As GPIHBP1 is not expressed on the endothelial cells in the brain, where glucose is the major fuel, GPIHBP1 exerts critical roles in delivering lipid nutrients to parenchymal cells^17^. The tracing study of GPIHBP1‐specific monoclonal antibody, 11A12, into a quadricep muscle^18^ and intravenous^19^ injection showed its bidirectional movement between the abluminal and luminal surface of capillary endothelial cells by a vesicular transport, as the transluminal transport was blocked by dynasore^19^.

**Figure 2:**
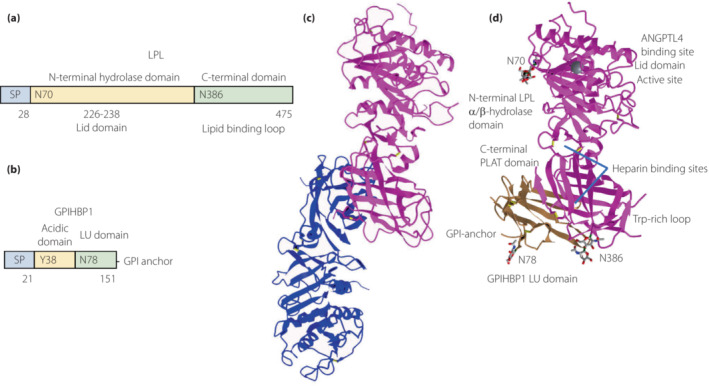
The structure of lipoprotein lipase (LPL) homodimer and LPL–glycosylphosphatidylinositol‐anchored high‐density lipoprotein binding protein 1 (GPIHBP1) complex. (a) N‐terminal hydrolase with Lid domain and C‐terminal domain with lipid‐binding loop of LPL. Signal peptide (SP) and N‐glycosylation sites (N70 and N386) are shown. (b) Acidic domain and cysteine‐rich LU (Ly6/uPAR) domain, and C‐terminal hydrophobic regions of GPI‐anchored protein are shown. Y38 is modified by sulfation, and N‐glycosylation site (N78) is shown. (c) Ribbon model structures of *Bos taurus* LPL homodimer are obtained from PDB ID: 8ERL at MMDB (NCBI). Head‐to‐tail homodimer formation was thought to be critical for LPL secretion and the maintenance of the enzymatic activity, whereas recent data suggested that LPL homodimer is present *in vitro*, and LPL is synthesized and secreted as a monomer *in vivo*. (d) Ribbon model structures of human LPL/GPIHBP1 complex (pink and brown) are obtained from PDB ID: 6E7K at MMDB (NCBI). N‐terminal LPL α/β‐hydrolase domain and C‐terminal PLAT (polycystin‐1, lipoxygenase, α‐toxin) domain of LPL and GPIHBP1 LU domain are shown. Portions of angiopoietin‐like protein (ANGPTL) binding site, the lid domain covering the catalytic active site, heparin binding sites and Trp‐rich loop in LPL are also shown. N‐terminal sequences (residues 21–61) containing the disordered acidic domain of GPIHBP1 are not defined in the ribbon diagram.

GPIHBP1 consists of the N‐terminal acidic domain rich in aspartates and glutamates, a three‐fingered cysteine‐rich LU (Ly6/uPAR) domain, and C‐terminal hydrophobic regions of GPI‐anchored protein. In the initial studies using expression of mutant GPIHBP1 in Chinese hamster ovary cells, the Ly6/uPAR (LU) domain is essential for the binding to LPL. The replacement of the LU domain of GPIHBP1 with that of CD59 and mutation of any of the 10 cysteines abolished the LPL binding^20^ (Figure 2). To date, hydrogen deuterium exchange mass spectrometry is used to investigate protein conformation, protein dynamics, and protein–ligand and protein–protein interface sites by detecting the reversible exchange between backbone amide hydrogen in a protein and deuterium from the solvents^21^. The binding between GPIHBP1 and LPL reduced hydrogen deuterium exchange in GPIHBP1 (amino acids 104–135) and LPL (429–446), and a synthetic peptide of GPIHBP1 (21–53) reduced deuterium uptake in LPL (306–320), suggesting a role for the acidic domain in LPL binding^22^. In the surface plasmon resonance studies, the acidic domain of GPIHBP1 increases the probability of a GPIHBP1–LPL encounter through electrostatic interaction compared with GPIHBP1 mutant lacking an acidic domain by increasing the association rate constant (*k*_*on*_) for LPL binding by >250‐fold^23^. By hydrogen deuterium exchange mass spectrometry study, full‐length GPIHBP1 reduces the spontaneous unfolding of LPL's hydrolase domain and preserves catalytic activity, whereas GPIHBP1 lacking the acidic domain has minimal ability to prevent unfolding^22^. Mice with the mutant allele, *Gpihbp1* ^*S/S*^, in which 17 residues in the acidic domain, including the sulfated tyrosine and the long stretch of acidic residues, were replaced with an S‐protein tag, showed elevated TG levels, lowered plasma LPL activity, marked reduction of GPIHBP1 and LPL on the luminal surface of the endothelial cells, and increased on the abluminal surface^24^. The series of the GPIHBP1 data is critical for transport of endothelial cells to the abluminal surface to the luminal surface, stabilization of enzymatic activity of LPL and LPL‐mediated TG‐rich lipoprotein processing.

## GPIHBP1 MUTATIONS IN SEVERE HYPERTRIGLYCERIDEMIA

Patients with missense mutations of GPIHBP1 have been reported, and they showed severe hypertriglyceridemia, recurrent acute pancreatitis and other complications (Table 1). Most of the mutations are located in the LU domain (63–139)^25^, ^26^, ^27^, ^28^, ^29^, ^30^, ^31^, ^32^, ^33^, ^34^, ^35^, ^36^, whereas p.C14F in signal peptide^37^, ^38^ and p.G175R close to the GPI‐anchored domain^27^ were also reported. In the LU domain, many of the mutations involve a cysteine^27^, ^28^, ^29^, ^32^, ^35^, which disrupts the C65‐C89 disulfide bond and causes major alteration of LPL binding. The folded LU domain is responsible for the formation of a stable complex with LPL, whereas the acidic domain stabilizes the hydrolase domain of LPL against spontaneous unfolding. The binding of GPIHBP1 to LPL was reduced in mutants with p.Q115P^26^, p.C89F^27^, p.C65Y^29^, p.C65S and p.C68G^32^. Although the binding between GPIHBP1 and LPL is intact, p.G175R, p.C14F and p.C14F/p.C68R variants reduce the cell surface expression of GPIHBPs^27^, ^38^. A 17.5‐kb deletion spanning the entire GPIHBP1 gene^27^, ^39^, a point mutation in intron 2 with skipping of exon 3^31^, and frame shift mutation c.48_49insGCGG (p.P17A fs*22)^40^ caused major defects of GPIHBP1 protein.

**Table 1:** Mutations in the glycosylphosphatidylinositol‐anchored high‐density lipoprotein binding protein 1 gene in patients with severe hypertriglyceridemia

Authors	*GPIHBP1*	Zygosity	Maximum triglyceride (mg/dL)	Complications
Ariza^25^	p.M1I	Homozygous	4,489	
	p.T80K	Homozygous	3,820	Acute pancreatitis
Beigneux^26^	p.Q115P	Homozygous	3,366	
Charriere^27^	p.C14F/p.C89F and deletion	Compound heterozygous	1,736	Acute pancreatitis
	p.G175R	Homozygous	2,303	Acute pancreatitis
Coca‐Prieto^28^	p.C68Y	Homozygous	1,398	Acute pancreatitis
Franssen^29^	p.C65Y	Homozygous	4,005	Acute pancreatitis
Gonzaga‐Jauregui^30^	p.T111P and p.V138fs	Compound heterozygous	12,030	Acute colitis
Lima^31^	c.182‐1G>T	Homozygous	885–20,600 (*n* = 12)	Acute pancreatitis (*n* = 4)
Lin^40^	p.P17A fs*22	Homozygous	1,514	Acute pancreatitis during pregnancy
Olivecrona^32^	p.C65S/p.C68G	Heterozygous	806–2,595 (*n* = 3)	Acute pancreatitis (*n* = 2)
Plengpanich^33^	p.S107C	Homozygous	3,164	Epigastric discomfort
Rios^39^	17.5‐kb deletion	Homozygous	>25,000	
Rodriguez^34^	APOA5 p.S232P and p.G175R	Compound heterozygous	1,015	
Surendran^35^	p.C65Y	Homozygous	>886	
	p.T108R	Homozygous	>886	Acute pancreatitis
	p.Q115P	Homozygous	>886	
	p.S144F	Heterozygous	>886	Acute pancreatitis
Wang^36^	p.G56R	Homozygous	4,252 and 7,095 (*n* = 2)	Acute pancreatitis (*n* = 2)
Xie^37^	LPL pA98T and p.C14F	Compound heterozygous	1,919	Acute respiratory distress syndrome
Yamamoto^38^	p.C14F and p.C68R	Homozygous	940	Acute pancreatitis

## AUTOANTIBODIES IN SEVERE HYPERTRIGLYCERIDEMIA

The acquired cases with severe hypertriglyceridemia caused by GPIHBP1 autoantibodies were discovered by the screening of the patients with unexplained hypertriglyceridemia^41^, ^42^. The plasma samples from the patients with unexplained hypertriglyceridemia and without mutations in LPL or GPIHBP1 at lipid clinics (*n* = 130), and six patients from unexplained hypertriglyceridemia, including three patients with systemic lupus erythematosus, were positive for GPIHBP1 autoantibodies, and they were complicated by severe hypertriglyceridemia and pancreatitis. GPIHBP1 autoantibodies block the LPL binding to GPIHBP1‐transfected Chinese hamster ovary cells, suggesting that GPIHBP1 autoantibodies functionally blocked LPL‐mediated processing of TRL, causing severe hypertriglyceridemia^41^. GPIGBP1 autoantibody syndrome was found in the patients with multiple sclerosis treated with interferon β1a, who developed severe hypertriglyceridemia and very low plasma levels of GPIHBP1 and LPL^43^. After interferon β1a therapy was stopped, the plasma triglyceride levels returned to normal, and GPIHBP1 autoantibodies were undetectable. In the case with recurrent episodes of severe hypertriglyceridemia and acute pancreatitis, intermittent hypertriglyceridemia was caused by intermittent appearance of GPIHBP1 autoantibodies. The patient was finally treated with rituximab and methylprednisolone, which resulted in the disappearance of GPIHBP1 autoantibodies and normalization of plasma TG levels^44^. To date, cases with autoimmune diseases, such as SLE^45^, ^46^, ^47^, antiphospholipid syndrome^46^ and Graves' disease^46^, ^48^, have been reported, and they were successfully treated with immunosuppressive treatments with prednisolone, rituximab and mycophenolate mofetil.

## CIRCULATING LEVELS OF LPL AND RELATED APOLIPOPROTEINS IN DIABETES

Pre‐heparin plasma or serum LPL concentrations were reduced in LPL‐deficient individuals, and they have been used for evaluation of patients with diabetes and obesity^49^. LPL mass was lower in patients with type 2 diabetes compared with individuals with normal glucose tolerance^50^. LPL mass showed significant positive relationships with serum HDL cholesterol and APOA1, and negative relationships with serum TG, insulin resistance and visceral fat area^49^. Reduced levels of serum LPL are associated with an increased risk for future cardiovascular disease in the EPIC‐Norfolk prospective population study^51^. Laparoscopic sleeve gastrectomy significantly increases serum LPL levels, and reduces body mass index and glycated hemoglobin levels compared with the nonsurgical treatments group^52^.

Although APOC2 is a critical factor for LPL activity and APOA5 stabilized LPL‐APOC2 complex, the reports of circulating levels of APOC2 and APOA5 as biomarkers in patients with type 2 diabetes are scarce. In type 2 diabetes patients with or without diabetic retinopathy, multivariate logistic regression analysis showed that APOC2/APOC3 and APOB/non‐HDL cholesterol, as well as APOE/APOC2, were independently related to the risk for the occurrence and severity of diabetic retinopathy^53^. Both APOC1 and APOC3 inhibit LPL activation, and the circulation levels are investigated in patients with type 2 diabetes. APOC1 concentrations were higher in patients with type 2 diabetes and significantly positively correlated with plasma TG levels, and not visceral adipose tissues^54^. APOC1 serum concentrations were also higher in patients with diabetic nephropathy (DN) compared with the healthy population^55^. The plasma APOC3 levels were also associated with higher TG levels and higher coronary artery calcification in patients with type 2 diabetes^56^. Glucagon‐like peptide‐1 agonists are known to improve the postprandial elevation of triglyceride and TRL. Treatment with liraglutide for 16 weeks in patients with type 2 diabetes showed the significant reduction of the secretion rate and plasma concentration of APOC3 after a fat‐rich meal shown by the injection of [5,5,5‐^2^H_3_]leucine and ultrasensitive mass spectrometry techniques^57^. Interestingly, in addition to type 2 diabetes, the association of APOC3 with insulin resistance, coronary artery calcium deposition^58^ and cardiovascular risk^59^ in patients with type 1 diabetes was also reported. In mouse models of type 1 diabetes‐accelerated atherosclerosis, relative insulin deficiency rather than hyperglycemia elevated levels of APOC3, and antisense oligonucleotide of APOC3 treatment abolished the increased hepatic APOC3 expression and lowered TRLs in diabetic mice without improving glycemic control^59^.

## CIRCULATING LEVELS OF ANGPTLS IN DIABETES AND OBESITY

ANGPTL 3, 4 and 8 inhibit the activity of LPL, and their circulating levels in the patients with type 2 diabetes have been reported. Plasma concentrations of ANGPTL4 were significantly increased in obese patients with impaired fasting glucose compared with lean participants. ANGPTL4 positively correlated with body mass index, waist circumference, fat mass, glycated hemoglobin, homeostatic model assessment of insulin resistance (HOMA‐IR), fasting TGs and inflammatory markers^60^. Similarly, increased plasma ANGPTL3 levels in type 2 diabetes patients were observed compared with non‐diabetic individuals^61^, and a positive correlation of ANGPTL3 with plasma glucose, insulin and HOMA‐IR was observed in insulin‐resistant states^62^. Plasma levels of ANGPTL8 were also higher in type 2 diabetes patients compared with non‐diabetic individuals^61^, and ANGPTL8 positively correlated with TG values^63^. Serum ANGPTL8 levels were also higher in individuals with gestational diabetes than the healthy control group, and positively correlated with fasting plasma glucose, HOMA‐IR and TG levels^64^. Serum levels of ANGPTL8 were significantly higher in DN compared with type 2 diabetes patients without DN. ANGPTL8 showed a positive correlation with high‐sensitivity C‐reactive protein, and a negative correlation with estimated glomerular filtration rate, but there was no significant correlation to HOMA‐IR^65^. The investigations of circulating apolipoproteins and ANGPTLs related to LPL in diabetes and obesity are important, as the inhibitors against APOC3 and ANGPTL3 using antibodies, antisense oligonucleotides and silencing ribonucleic acids are currently under development^66^.

## CIRCULATING LEVELS OF GPIHBP1 AND DIABETIC VASCULAR COMPLICATIONS

Although GPIHBP1 is an important co‐factor for the maintenance of the activity and localization of LPL on the luminal surface of capillary endothelial cells, the circulating GPIHBP1 levels and their significance are not well understood, as an enzyme‐linked immunosorbent assay for GPIHBP1 was not available. In 2018, a sandwich enzyme‐linked immunosorbent assay was developed^67^. By using the enzyme‐linked immunosorbent assay, GPIHBP1 was undetectable in the plasma of individuals with null mutations in GPIHBP1^67^, and the plasma levels of GPIHBP1 were very low in the patients with GPIHBP1 autoantibody syndrome^43^. Serum GPIHBP1 median levels were 849 pg/mL in healthy volunteers and 1,087 pg/mL in patients with a history of cardiovascular or metabolic disease^67^. Serum GPIHBP1 levels were higher in patients with type 2 diabetes compared (952.7 pg/mL) with non‐diabetic individuals (700.6 pg/mL)^68^. Unexpectedly, there were no differences in serum GPIHBP1 levels in type 2 diabetes patients with and without hypertriglyceridemia, whereas serum GPIHBP1 levels were significantly higher in patients with type 2 diabetes with diabetic retinopathy, DN and microvascular complications than in those without these complications. The cleavage and release of GPIHBP1 from endothelial cells to the circulation might be mediated by glycosylphosphatidylinositol‐specific phospholipase D, an enzyme that specifically cleaves glycosylphosphatidylinositol anchors^69^, or secretion of exosome containing GPIHBP1^70^. Further studies are required to ascertain the significance of GPIHBP1 as a biomarker and therapeutic target in the management of obesity, diabetes, and dyslipidemia.

GPIHBP1 is a crucial molecule for the translocation of LPL from parenchymal cells to the luminal surface of capillary endothelial cells and maintenance of lipolytic activity. The null mutation of GPIHBP1 and the production of GPIHBP1 autoantibody induce severe hypertriglyceridemia and recurrent episodes of acute pancreatitis. In patients with type 2 diabetes, the circulating GPIHBP1 levels might not correlate well with TRL, and rather well‐reflect the microvascular complications, such diabetic retinopathy and diabetic kidney disease.

## DISCLOSURE

Jun Wada receives speaker honoraria from Astra Zeneca, Bayer, Boehringer Ingelheim, Daiichi Sankyo, Kyowa Kirin, Novo Nordisk and Mitsubishi Tanabe, and receives grant support from Bayer, Chugai, Kyowa Kirin, Otsuka, Shionogi, Sumitomo and Mitsubishi Tanabe. Jun Wada is an Editorial Board member of *Journal Of Diabetes Investigation* and he is excluded from all editorial decision‐making related to the acceptance of this article for publication.
